# Right ventricular outflow tract obstruction by cardiac hemangioma in asymptomatic patient

**DOI:** 10.1093/jscr/rjae321

**Published:** 2024-05-18

**Authors:** Naritsaret Kaewboonlert, Piyapat Chunharas, Naree Pluthikarmpae, Jiraphon Poontananggul, Akharawat Wongthep, Natthipong Pongsuwan, Udomsak Lerssuttipon

**Affiliations:** Institute of Medicine, Suranaree University of Technology, Nakhon Ratchasima 30000, Thailand; Department of Medicine, Maharat Nakhon Ratchasima Hospital, Nakhon Ratchasima 30000, Thailand; Department of Pathology, Suranaree University of Technology Hospital, Nakhon Ratchasima 30000, Thailand; Institute of Medicine, Suranaree University of Technology, Nakhon Ratchasima 30000, Thailand; Institute of Medicine, Suranaree University of Technology, Nakhon Ratchasima 30000, Thailand; Institute of Medicine, Suranaree University of Technology, Nakhon Ratchasima 30000, Thailand; Department of Medicine, Maharat Nakhon Ratchasima Hospital, Nakhon Ratchasima 30000, Thailand

**Keywords:** cardiac tumor, ventricular hemangioma, right ventricular outflow tract obstruction, benign tumor

## Abstract

Ventricular hemangiomas are rare benign tumors, pose diagnostic and therapeutic complexities. We report a case of a 52-year-old female with essential hypertension who developed a systolic ejection murmur during a hypertension clinic visit. The echocardiogram revealed a hyperechoic mass obstructing the right ventricular outflow tract, causing enlargement of the right atrium and ventricle, with a reduction in the right ventricular ejection fraction. Due to the risk of death, the patient underwent an emergency surgical resection along with tricuspid valve replacement. Postoperative recovery was uneventful, and subsequent cardiac magnetic resonance imaging showed an improvement in ejection fraction without residual tumor. This case highlights the diagnosis and therapeutic complexities of ventricular hemangiomas. With this report, we aim to provide a comprehensive review of ventricular hemangiomas and to enhance understanding of this condition for improved patient care.

## Introduction

Primary cardiac tumors are rare, with a prevalence of ~0.0102% to 0.056% in autopsy series [1, 2]. Among them, ventricular hemangiomas constitute ~2.8% to 4.5% cases in the reported case series [3, 4]. Various imaging modalities provide valuable insights on tumor characteristics, including size, location, vascularity, and impact on adjacent cardiac structures [5]. The management of ventricular hemangiomas remains a topic of debate due to their rarity and limited clinical evidence. Surgical resection is often considered the gold standard, but it can be challenging when tumors infiltrate critical cardiac structures. Alternative approaches, such as medical treatment with propranolol, have been reported, but their efficacy and long-term outcomes are uncertain [6].

In this report, we present a case of right ventricular hemangioma obstructing the ventricular outflow tract and causing an impairment of the right ventricular function. Written informed consent was obtained from the patient for publication of this report. The study was approved by the institutional ethical committees (EC-65-0116).

## Case report

A 52-year-old female with essential hypertension was prescribed atenolol and hydrochlorothiazide/amiloride. During a follow-up visit at the hypertension clinic, a systolic ejection murmur grade IV/VI with a systolic thrill at the left parasternal border was detected. She denied experiencing orthopnea or paroxysmal nocturnal dyspnea and had neither history of weight loss nor palpitations. There was no notable history of cancer in her family.

Transthoracic echocardiogram revealed a hyperechoic mass within the right ventricular cavity, causing right outflow tract obstruction (a maximum velocity of 4.2 m/s and a pressure gradient across the right ventricular outflow tract of 73 mmHg). The right atrial and the right ventricular chambers appeared dilated, while the left ventricular ejection fraction was preserved without regional wall motion abnormality.

**Figure 1:**
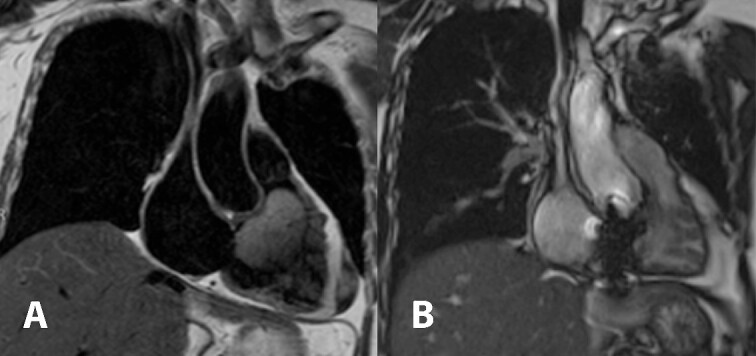
Cardiac magnetic resonance imaging demonstrated T2 hyperintense partially mobile 60 × 39 × 46 mm mass occupying the right ventricular cavity and causing outflow tract obstruction (A). Comparison to post-surgical resection in picture (B) showed decreased right atrial and ventricular size, the tricuspid bioprosthetic valve in place, and right ventricular function returned to normal.

Cardiac magnetic resonance imaging showed a partially mobile mass measuring 60 × 39 × 46 mm within the right ventricular cavity. The mass is isosignal on T1-weighted images, hyperintense signal on T2-weighted images (Fig. 1A), and hyperintense signals on T2 fat-suppression sequences. It obstructed the right ventricular outflow tract, abutting the right ventricular wall without definite invasion, likely extending from the right atrium, although a stalk was unidentified. First-pass perfusion imaging indicated a normal myocardial perfusion pattern with heterogeneous vivid enhancement of the mentioned mass. Tricuspid valve regurgitation was observed, and the right ventricular ejection fraction was 14% while the left ventricular ejection fraction was 70%.

**Figure 2:**
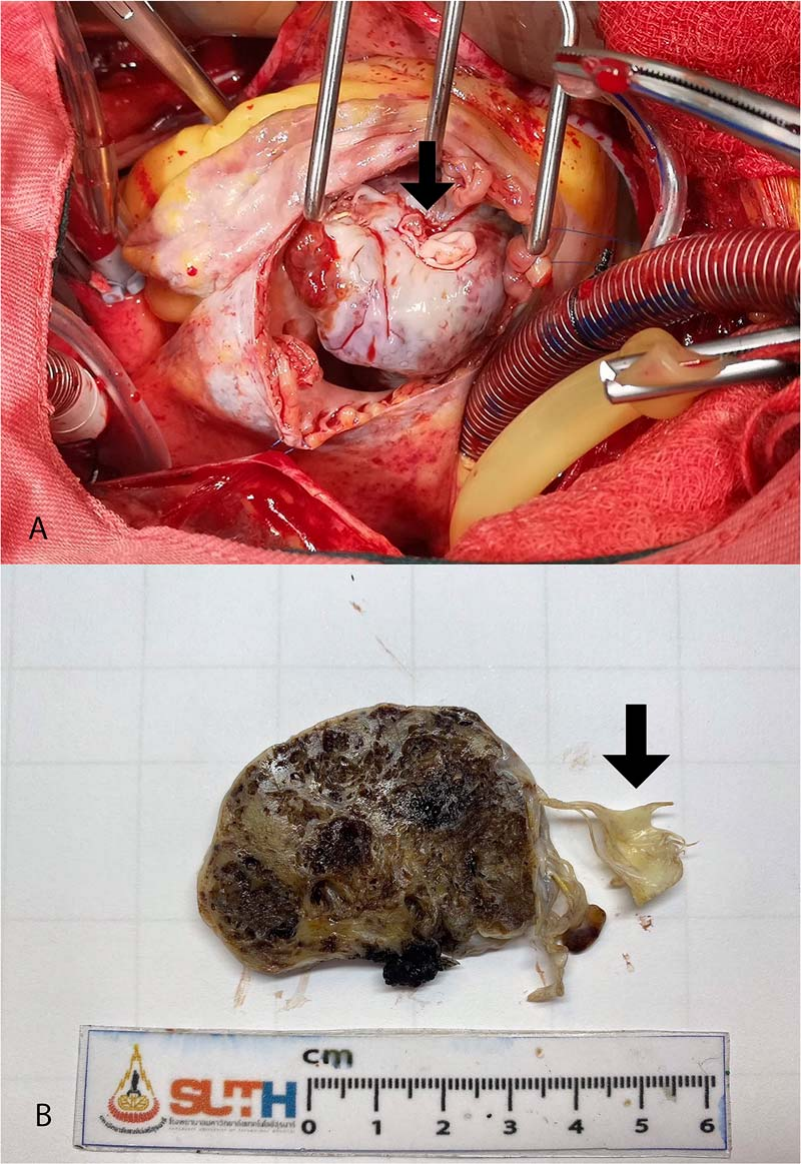
Intra-operative finding shows lobulated mass protruding from right atriotomy involving the anterior leaflet of the tricuspid valve (A). The gross pathological cut surface shows a polypoid mass with spongy appearance and focal fibrosis (B). Arrows are indicated anterior leaflet of the tricuspid valve.

The surgical resection was performed under total cardiopulmonary bypass while inducing cardiac arrest. Accessed by right atriotomy and right ventriculotomy, respectively (Fig. 2A). The tumor originated from the right ventricular surface beneath the base of the anterior leaflet of the tricuspid valve. It extended into the right ventricular outflow tract and was attached to the anterior leaflet and head of the anterior papillary muscle (Fig. 2B). The tumor and tricuspid valve were successfully removed. The tricuspid valve was replaced with a 31-mm bioprosthetic valve. The patient was discharged home on the fifth day after the surgery.

**Figure 3:**
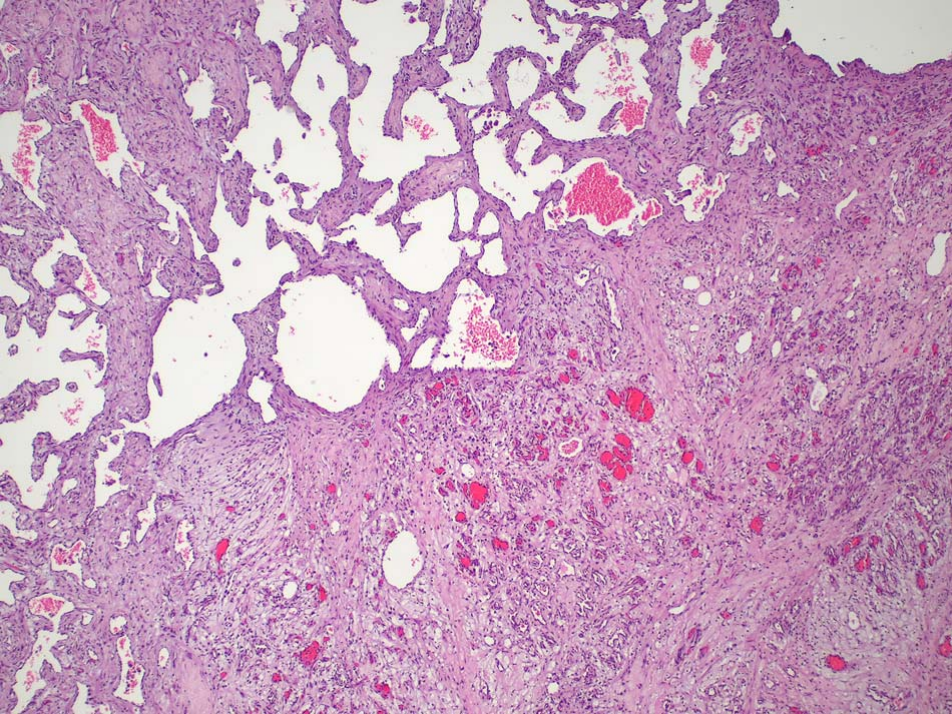
Mixed cavernous-capillary hemangioma. The histologic examination reveals dilated thin-walled blood vessels (left upper) admixed with capillary-sized blood vessels (right lower) on fibrotic stroma (hematoxylin and eosin, 40x).

The pathology report confirmed the diagnosis of a mixed cavernous-capillary type cardiac hemangioma (Fig. 3). The gross pathological cut surfaces showed polypoid mass with a spongy light-to-dark brown appearance and focal fibrosis. The tumor involved tricuspid valve and resection margin (Fig. 2B). The histology showed the proliferation of capillary-sized blood vessels admixed with dilated thin-walled blood vessels. The vascular channels were lined by flat to plump endothelium. No nuclear pleomorphism, mitosis, or tumor necrosis was identified (Fig. 3).

In the follow-up visit on the fourth month after the operation, cardiac magnetic resonance imaging was obtained and showed a significant improvement of the right ventricular ejection fraction from 14% to 68%. There was also a noticeable decrease in the size of the right ventricle. The tricuspid prosthetic valve appeared to have normal function, and no residual tumor was detected (Fig. 1B).

## Discussion

Ventricular hemangiomas are characterized by an abnormal proliferation of blood vessels within the myocardium or cardiac chamber. Clinical manifestations depend on size and location of the tumor. Patients may either remain asymptomatic or present with a wide range of symptoms, including palpitations, chest pain, dyspnea, syncope, or arrhythmias [5].

Transthoracic echocardiography is usually the first study performed in patients with cardiac symptoms and serves as an important evaluation for postoperative care [7]. Cardiac magnetic resonance imaging plays an important role in understanding tumors based on specific signal patterns seen using T1 and T2 weights [8].

Management of ventricular hemangioma remains a challenging topic due to their rarity and limited clinical evidence. The treatment approach depends on several factors, including tumor size, symptoms, growth pattern, and the potential risk of complications. While surgical resection is a standard treatment with an operative mortality rate of 2% [9]. Tumor histology is the most significant predictor for mortality [9]. Alternative approaches, such as medical treatment with propranolol, due to having been established for a long time in the treatment of skin hemangioma, have been reported [10]. However, their efficacy and long-term outcomes are uncertain.

The diagnosis is essential to consider a differential diagnosis of more common cardiac tumors, including myxoma, fibroma, and angiosarcoma. Magnetic resonance imaging characteristics can be used to predict the likelihood of malignancy of a cardiac mass, including tissue heterogeneity on T1- and T2-weighted images, the presence of hemorrhage and necrosis within the mass, and contrast enhancement [8].

Complete tumor resection is crucial for curative treatment, but the natural history of ventricular hemangioma is not well understood. In cases, where the tumor is unresectable or has low chance of malignancy, long-term stability without obstruction or compression of adjacent structures has been observed [11]. Hemangiomas can be treated with beta-blockers, which have shown a decrease in tumor volume [10, 12]. Heart transplantation is considered as a last resort for the treatment of primary non-operable benign cardiac tumors [13].

## Conclusion

Ventricular hemangiomas are very rare primary cardiac tumor with challenging management due to limited evidence on effective treatment strategies. This case underlines the importance of thorough diagnostic imaging for surgical planning. Complete resection of the tumor resulted in remarkable improvement in the patient’s cardiac function and surveillance for tumor recurrence is conducted.
